# Currarino’s triad: Intraoperative ultrasound image-guided surgery

**DOI:** 10.4103/0971-9261.72438

**Published:** 2010

**Authors:** C. R. Thambidorai, Latif K. E. Adbel, A. Zulfiqar

**Affiliations:** Department of Surgery, University Kebangssan Malaysia Medical Centre, Kuala Lumpur, Malaysia; 1 Department of Radiology, University Kebangssan Malaysia Medical Centre, Kuala Lumpur, Malaysia

**Keywords:** Currarino’s triad, excision, intra-operative ultra-sound, pelvic mass

## Abstract

This is a report on the use of transperineal intraoperative ultrasound imaging in a case of Currarino’s triad for the first time in the literature.

## INTRODUCTION

The syndrome consisting of anorectal malformation, sacral bone abnormality and presacral mass is known as Currarino’s triad. Chronic constipation is the most common presenting feature of this syndrome.[1] The sacral anomaly is typically sickle shaped and is known as scimitar sacrum. The presacral mass may be a meningocele (in nearly half the cases) or a teratoma or dermoid cyst.[1] Magnetic resonance imaging (MRI) is considered the investigation of choice for preoperative evaluation of Currarino’s triad to detect the presacral mass and any anomalies of the spinal canal.[2]

At surgery, precise dissection of the presacral mass is required in view of its deep location, adherence to the rectal wall, proximity to the pelvic nerves and, often, extension into the intrathecal space. Transperineal intraoperative ultrasound (IOUS) imaging was used in a boy with Currarino’s triad to aid precise definition of the extent of the pelvic mass and its intraspinal extension. Use of IOUS for Currarino’s triad has not been reported before.

## CASE REPORT

**Figure 1:**
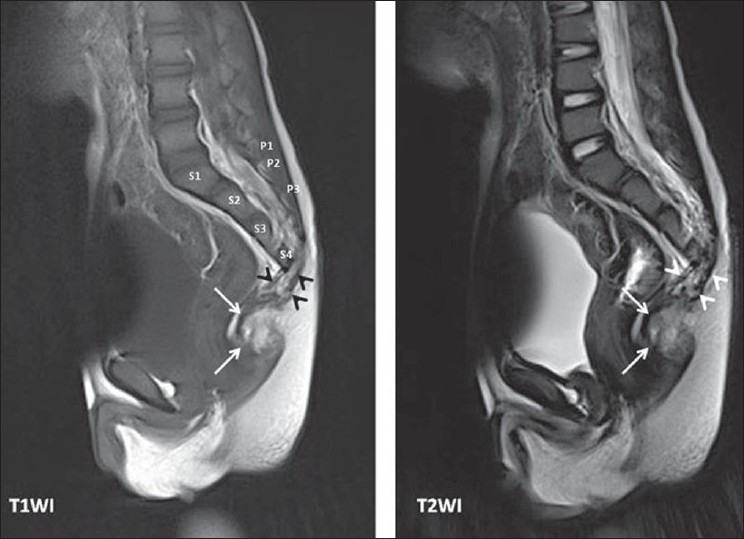
Sagittal T1-weighted and T2-weighted images show the mass (arrows) that extends into the spinal canal (arrow heads). S1, S2, S3 and S4 denote the body of the four sacral vertebrae and P1, P2 and P3 denote the posterior elements of the first to third sacral vertebrae in the T1 image

A 3-year-old boy presented with abdominal distension and history of constipation, the latter since early infancy. The abdomen was distended, with palpable fecal masses, and the rectal examination revealed a stenosed anus. The abdominal radiograph showed dilated bowel loops with deformed sacrum. The patient was initially treated with stool softeners and rectal washouts. Rectal biopsy showed normal ganglion cells. Pelvic MRI showed hypoplasia of body of the fourth sacral vertebra with absence of its posterior elements and absence of whole of the fifth sacral vertebra. A presacral mass measuring about 3 cm × 3 cm × 3 cm and consisting mainly of fat with locules of fluid was seen distal to the fourth sacral vertebra with intraspinal extension [Figure 1].

**Figure 2:**
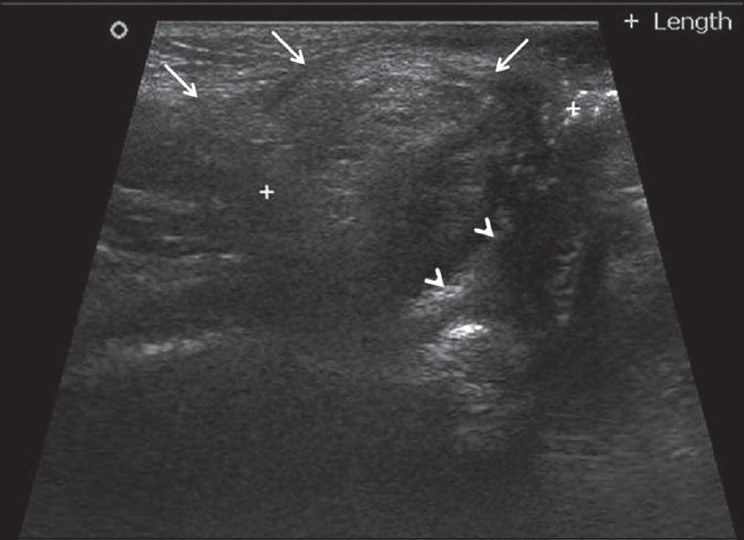
Ultrasound of the perineum shows the mass (arrows) posterior to the rectum (arrow heads)

Divided transverse colostomy was performed initially and 3 months later and definitive surgery was performed through a posterior sagittal perineal approach. Transperineal IOUS imaging with a 7.5 MHz linear transducer placed within the gluteal cleft was used to define the anatomy of the pelvic mass during excision [Figure 2]. The intrathecal prolongation was also excised. Completeness of excision of the mass was confirmed both visually and by IOUS. The narrowed anal canal was mobilized and the rectum above the stenosis was brought down to the site of the old anus with reconstruction of the sphincter muscles. The postoperative period was uneventful.

**Figure 3:**
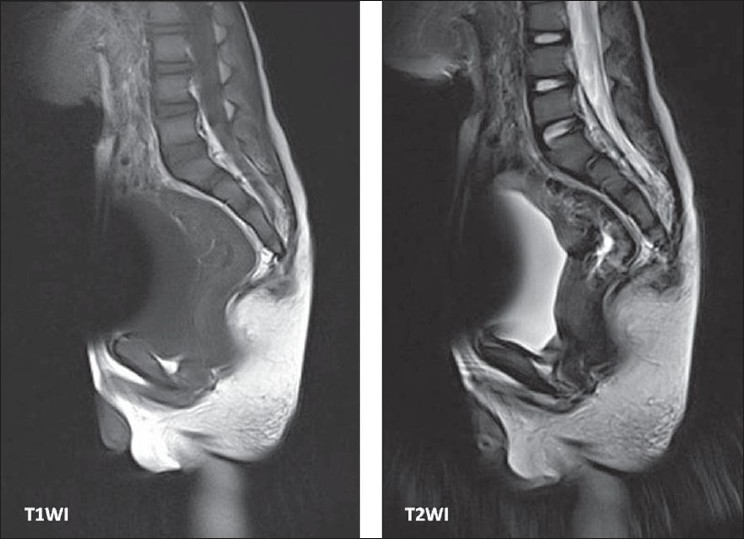
Postoperative sagittal T1-weighted and T2-weighted images

Histological examination of the pelvic mass revealed a mature teratoma with clear surgical margins. MRI performed 4 months later showed no residual tumor [Figure 3]. The colostomy was closed. Currently, he passes stool once in 1–3 days without laxatives and is totally continent.

## DISCUSSION

In surgery for tumors of the liver, pancreas, biliary tree and pelvis, IOUS imaging has been used extensively for the diagnosis of impalpable lesions, to locate multiple lesions, for precise delineation of adjacent structures such as vessels and nerves and also to confirm completeness of excision.[3] However, use of IOUS in Currarino’s triad in a child has not been reported previously.

Transperineal IOUS guidance is a useful tool in the precise surgical management of Currarino’s triad and is easy to organize with the help of a radiologist or with some training by the surgeon concerned. Transrectal IOUS (although likely to provide clearer images than transperineal IOUS) is not feasible in most children with Currarino’s triad because of the anorectal stenosis and the relatively large size of transrectal probes currently available.
